# Prediction of Tail Biting Events in Finisher Pigs from Automatically Recorded Sensor Data

**DOI:** 10.3390/ani9070458

**Published:** 2019-07-19

**Authors:** Mona Lilian Vestbjerg Larsen, Lene Juul Pedersen, Dan Børge Jensen

**Affiliations:** 1 Department of Animal Science, Aarhus University, Blichers Allé 20, DK-8830 Tjele, Denmark; 2 Department of Veterinary and Animal Sciences, University of Copenhagen, Grønnegårdsvej 2, DK-1870 Frederiksberg C, Denmark; 3 Department of Large Animal Sciences, University of Copenhagen, Grønnegårdsvej 2, DK-1870 Frederiksberg C, Denmark

**Keywords:** Sus scrofa domesticus, precision livestock farming, computational ethology, drinking behaviour, water flow, pen temperature, dynamic linear models, artificial neural network, Bayes’ Theorem, Bayesian ensemble

## Abstract

**Simple Summary:**

Tail biting is a major animal welfare issue within modern pig production, and tail biting should be prevented whenever possible. If the farmer could get an alarm when a pen of pigs is at high risk of developing tail damage, the farmer would be able to take timely action to prevent tail damage in specific pens. In the current investigation, a method for prediction of tail biting events was developed and tested in a real-life setting. The method used changes in pigs’ drinking behaviour and in the temperature of the pen. The method was able to alarm the farmer about 12 of the 14 tail biting events prior to serious tail damage. However, the farmer did also get false alarms on 30% of the days without tail biting events, which is not optimal. Thus, the farmer could use the alarms as indications of which pens to pay greater attention to. The next step could be to expand the method to include behavioural changes that are more specific to tail biting such as changes in the pigs’ tail posture.

**Abstract:**

Tail biting in pigs is an animal welfare problem, and tail biting should be prevented from developing into tail damage. One strategy could be to predict events of tail biting so that the farmer can make timely interventions in specific pens. In the current investigation, sensor data on water usage (water flow and activation frequency) and pen temperature (above solid and slatted floor) were included in the development of a prediction algorithm for tail biting. Steps in the development included modelling of data sources with dynamic linear models, optimisation and training of artificial neural networks and combining predictions of the single data sources with a Bayesian ensemble strategy. Lastly, the Bayesian ensemble combination was tested on a separate batch of finisher pigs in a real-life setting. The final prediction algorithm had an AUC > 0.80, and thus it does seem possible to predict events of tail biting from already available sensor data. However, around 30% of the no-event days were false alarms, and more event-specific predictors are needed. Thus, it was suggested that farmers could use the alarms to point out pens that need greater attention.

## 1. Introduction

One of the challenges experienced by pig producers is how to prevent the pigs from biting the tail of other pigs, also referred to as tail biting, without resorting to tail docking. Tail biting is painful for the bitten pigs [1] and may result in infections [2], thus lowering the welfare of the bitten pigs. Further, tail biting is a sign of frustration in the tail biter and perhaps general stress in the pen, as many risk factors of tail biting are at pen level [3]. Thus, tail biting may be a sign of lowered welfare for all pigs in the pen. Tail biting is also an economic problem for the farmer due to the costs of medical treatments, decreased growth rate [4], loss of pigs and carcass condemnations at the abattoir [5,6]. Lastly, according to EU legislation, farmers cannot routinely tail dock their pigs and this should theoretically be a last resort (EU Council Directive 2008/120/EC).

Tail biting is a multifactorial problem, and thus general risk reduction can be a challenge and may not be enough to lower the incidence of tail biting in undocked pigs to an acceptable level [7]. A supplementary strategy is early detection, using sensor data to predict pens showing early signs of tail biting and making it possible for the farmer to make timely interventions. Unfortunately, the sensor data currently easily available in a pigpen, although not yet used in normal practice, are limited to feed usage, water usage and temperature at pen level. Changes in pigs’ drinking pattern has previously been hypothesised to be a sign of stress [8], and changes in both water usage and pen temperature can represent a behavioural change. Pigs’ daily pattern in drinking behaviour seems to follow the daily pattern in activity [9] which has formerly been shown to change prior to tail biting [10]. Also, pigs seem to increase their exploratory behaviour prior to tail biting towards other parts of the pen than pen mates [10], and this may also be directed towards the drinking nipple. The pen temperature seems to change as a reaction to pigs’ changes in lying location in the pen. This was seen prior to pen fouling where a decrease in pen temperature above the solid floor was found simultaneously with a decrease in pigs lying in the solid floor area [11]. Further, changes in pen temperature could also be a risk factor of tail biting. Thus, both sensor data on water usage and pen temperature may hold predictive value for tail biting. 

The aim of the current study was to investigate whether sensor data on water usage (water flow and activation frequency) and pen temperature (above the solid and slatted floor) had predictive value to tail biting. This was tested by describing the pattern in each data source, developing and optimising artificial neural networks (ANN) for each data source on parts of the data and testing the predictive performance of each ANN on the rest of the data. This was both done separately for each data source and combined using a Bayesian ensemble strategy. 

## 2. Materials and Methods 

### 2.1. Data Sources

Data used in the current study originate from four batches of finisher pigs raised from 30 kg to approximately 110 kg (slaughter) over 10 weeks at the experimental facilities at the Department of Animal Science, Aarhus University. The setup and collection of data for this study has been described in detail by Larsen et al. [7] and was approved by the Danish Animal Experiments Inspectorate (Journal no. 2015-15-0201-00593) prior to conduction. The study included 112 finisher pens (batches 1, 3 and 4: 32 pens each; batch 2: 16 pens) randomly assigned to one level of each of three treatments: (1) TAIL, pens with docked (*n* = 60) or undocked (*n* = 52) pigs; (2) STRAW, pens with no straw provided (*n* = 56) or provided with 150 g of straw per pig and day on the solid floor (*n* = 56); (3) SPACE, pens with a low (0.73 m^2^/pig, 18 pigs, *n* = 56) or high space allowance (1.21 m^2^/pig, 11 pigs, *n* = 56). The tails were docked according to Danish legislation within the first four days after birth with a hot-iron cutter to half of the tail’s original length. Straw was provided daily between 1000 and 1200 h. Both space allowances were above the EU legislative demand of 0.65 m^2^/pig for 110 kg pigs (EU Council Directive 2008/120/EC), but resembled what is practised in countries that banned tail docking (e.g., Sweden: 1.02 m^2^/pig, [12]).

**Figure 1:**
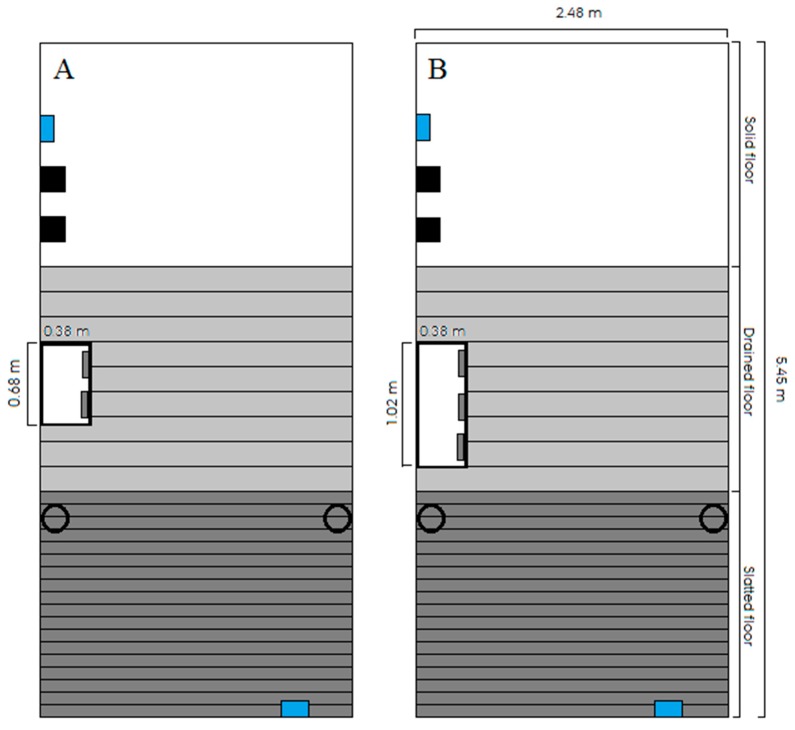
Drawing of pen dimension and design for (**A**) pens with a high space allowance of 1.21 m^2^/pig (11 pigs) and (**B**) pens with a low space allowance of 0.73 m^2^/pig (18 pigs). The white rectangle represents the feeder, and the solid black squares represent two wooden beams in separate vertical racks. The hollow, black circles represent drinking cups, while the blue rectangles represent temperature sensors. All pens had the same dimensions.

The design of the pens can be seen in Figure 1. The floor of the pens was divided between one third of solid, drained and slatted flooring. The gap between the slats was 2 cm for both the drained and slatted floor, whereas the slats were 8 cm wide for the slatted floor and 18 cm wide for the drained floor. The temperature curve used by the automated ventilation system to adjust the climate according to the weight of the pigs (SKOV A/S, Roslev, DK) decreased from 21 °C in week 1 after insertion to 17 °C in week 8 and onwards. Each pen further included an automatically controlled sprinkler system (SKOV A/S, Roslev, DK) above the slatted floor. The pigs were fed ad libitum with a commercial dry feed (15.1–15.5% crude protein), and the feeder was filled each day at 0300 h, 1000 h and 1830 h. Each pen included one dry feeder with either three or two feeding spaces, depending on the initial group size, separated by solid sides. Artificial light was on from 0530–1830 h. 

Each pen included two drinking cups, each with a liquid flow sensor (RS PRO Radialturbine Flowmåler, RS PRO, RS Components A/S, Copenhagen, Denmark, https://dk.rs-online.com/web/), connected to a system recording the pulses emitted from the sensor continuously every second. If no pulse was recorded within a specific second, the system noted this as a stop in water flow. When pulses were again recorded by the system, this was noted as a start in water flow. From these recordings, the water flow (L) and activation frequency (number of start recordings) per hour for each sensor were extracted. Prior to analysis, water flow and activation frequency were summed over the two liquid flow sensors per pen. Each pen also included two temperature sensors (as part of the climate control system of SKOV A/S, Roslev, DK, USA): one 63 cm above the solid floor and one 53 cm above the slatted floor, both placed on pen walls. Pen temperature was recorded every second but was aggregated to the average temperature (°C) for each hour and sensor prior to analysis.

Each day of the study, tail damage was recorded as an indicator of tail biting by the trained staff from outside the pen. Further, tail damage was recorded in more detail three times per week by trained technicians and researchers by entering the pen and looking at each individual tail. If at least one pig in the pen was observed with a bleeding tail wound, the pen was recorded as having a tail biting event (day 0). The pigs with bleeding tail wounds were moved to sick pens, and the detailed recording of tail biting was terminated for the particular pen, while the daily recording by the staff continued throughout the study. Thus, a pen could have multiple tail biting events throughout the study period, but only the first event for each pen was used to develop the prediction algorithm. At the same time as the recording of tail biting events, the trained staff also recorded fouling and diarrhoea events daily from outside the pen. A pen was recorded as experiencing a fouling event when at least half of the solid floor was wet with excreta, and as experiencing a diarrhoea event when at least one spot of diarrhoea was found in the pen.

In the current study, batches 1, 2 and 3 were used as the training data, whereas batch 4 was used as the test data. Thus, the development of the dynamic linear models (DLM) and the optimisation of the ANNs included data from batches 1, 2 and 3 only. Data from batch 4 were used to evaluate the performance of the final prediction algorithms.

Prior to analysis, the data sources water flow and activation frequency were square root transformed to better follow a Gaussian distribution.

### 2.2. Modelling of Linear Trend and Diurnal Pattern to Obtain Forecast Errors

All modelling and prediction algorithm development and evaluation were performed in the statistical language R [13].

The values from the four data sources (water flow, activation frequency, pen temperature above the solid floor, pen temperature above the slatted floor) were modelled using separate DLMs with a one-step Markov evolution [14]. The parameters of the DLMs were estimated using data from batches 1, 2 and 3, using only the pens that were not recorded to have a tail biting event throughout the growth period of the given batch. This selection had the purpose of defining the DLMs in such a way that they were optimised for describing the expected pattern of values from each data source under the assumption that the pigs are not experiencing problems such as tail biting; thus large deviations from the expected pattern, i.e., large forecast errors, would be indicative of such problems. 

In general, a DLM consists of an observation Equation (1) and a system Equation (2):

(1) $$Y_{t}={F^{\prime }}_{t}\theta_{t}+v_{t},\;v_{t}\sim N\left(\underline{0},V\right)$$

(2) $$\theta_{t}=G_{t}\theta_{t-1}+w_{t}\;w_{t}\sim N\left(\underline{0},W\right)$$

where $Y_{t}$ is the value of the data source at time *t*, $\theta_{t}$ is the unobservable parameter vector at time *t*, ${F^{\prime }}_{t}$ is the design matrix, $G_{t}$ is the system matrix, V is the observational variance, and W is the systematic co-variance matrix describing the co-variance between the systematic evolution of the observed variables and the systematic evolution of their linear trend components. In the current study, the time interval between observations was 1 h. The observation equation describes how the observed values of the data source depend on the parameter vector, whereas the system equation describes how the parameter vector is updated between adjacent hours. At each observation time *t*, the DLM produces a forecast for the observation value and an estimate of uncertainty for this forecast, expressed as the forecast variance. Based on the error of the forecast, $e_{t}$, the value of the forecast variance, $Q_{t}$, and the observational and systematic variances, the parameter vector undergoes a Bayesian update by means of the Kalman filter as described in detail by West & Harrison [12]. Furthermore, the value of the forecast error can be standardised as $u_{t}=e_{t}/\sqrt{Q_{t}}$.

In our study, the observational variance was assumed constant and was set to a certain percentage of the mean value of the data source. This percentage was optimised for each data source so that the resulting standardised forecast errors approximately followed a standard Gaussian distribution. The level of flexibility in the DLM for each data source was included by using a discount factor with a value between 0 and 1, with a higher value indicating less flexibility. The discount factor was optimised for each data source to the value resulting in the lowest root mean squared error and lowest mean absolute error by trying values between 0.8 and 1.0 by steps of 0.01.

In our study, the values of each of the four data sources were modelled as the sum of a linear trend and a diurnal pattern, following the example of Madsen et al. [15]. The diurnal pattern of the water flow and activation frequency was further modelled as the sum of three separate harmonic waves with one, two and three peaks per 24 h, respectively, as was done by Madsen et al. [15]. The diurnal pattern of the two temperature variables was also modelled as the sum of three separate harmonic waves with one, two and three peaks per 24 h, respectively. For the DLM implementation, we made use of the sine-cosine form of the function for the harmonic wave as seen in Equation (3):

(3) $$f\left(t\right)=\beta_{2}\times \cos\left(N\omega t\right)+\beta_{1}\times \sin\left(N\omega t\right)$$

where N in our case is the number of wave peaks per 24 h and ω=(2π)/24. For the DLM implementation, the parameter vector, $\theta_{t}$, for each variable would contain the expected initial level of the said variable, the expected linear growth per hour, and the $\beta_{2}$ and $\beta_{1}$ values for each of the three waves. The initial values of the parameter vector, $\theta_{0}$, for each data source were estimated by a Gaussian linear mixed model using the R function ‘lmer’ from the ‘lme4’ package [16]. Thus, $\theta_{t}$ was a column vector with a length of eight for all four data sources. The system matrix, $G_{t}$, was a block-diagonal matrix which contained a 2 × 2 block for updating the parameter vector, according to the initial mean and linear trend, as well as one 2 × 2 block per wave for updating the parameter vector according to the sine-cosine function as previously shown by Madsen et al. [13]. 

In addition to the sine-cosine form, a harmonic wave can also be described by an amplitude, *A*, and a phase shift, *c*, which is more intuitively interpretable. For this reason, the model estimates related to the harmonic waves were transformed to this form before being presented as results. This transformation was done using Equations (4) and (5):

(4) $$A=\sqrt{\beta_{1}{}^{2}+\beta_{2}{}^{2}}$$

(5) $$c=\mathrm{atan}2\left(\beta_{2},\beta_{1}\right)$$

Additionally, a third variable, b, is used in this form to express the frequency of the waves, which in our case is simply determined by whether the wave has 24-, 12- or 8-h cycles. 

The optimised DLMs were applied to data from all pens during batches 1 through 4, and the standardised forecast errors for each hour of observation were extracted. These standardised forecast errors from batches 1, 2 and 3 were then used in the optimisation of the ANNs. This optimisation is described in detail in Section 2.3.2.

### 2.3. Development of The Prediction Algorithms

To predict events of tail biting, the prediction algorithm should first be able to recognise pens with a tail biting event from pens without the event. Second, it should also be able to recognise days with a tail biting event from days without the event for the same pen. The development of the prediction algorithm focused on the first step. For this purpose, the pens with at least one event of tail biting (event pens) were paired with control pens from the same batch with the same levels of straw and space allowance treatments and that had not been scored with a tail biting event throughout the study period. Further, the standardised forecast errors were only extracted for the last 3 days prior to the first event day (day 0) for both the event and control pens (day-3, day-2 and day-1) for the purpose of the ANN optimisation.

#### 2.3.1. Evaluation Measures

The two prediction methods used in the current study (described in Section 2.3.2 and Section 2.3.4) both output a numerical value (probability) between 0 and 1. This value is then categorised based on a set or optimised threshold value. The following measures (Equations (6)–(9)) were used in the optimisation of the ANNs and fixed probability model and in the performance evaluation of the prediction algorithms, based on the categorised predictions:

(6) $$\mathrm{Sensitivity}=\frac{\mathrm{TP}}{\mathrm{TP}+\mathrm{FN}}$$

(7) $$\mathrm{Specificity}=\frac{\mathrm{TN}}{\mathrm{TN}+\mathrm{FP}}$$

(8) $$\mathrm{Alarm}\;\mathrm{error}\;\mathrm{rate}=\frac{\mathrm{FP}}{\mathrm{TP}+\mathrm{FP}}$$

(9) $$\mathrm{Accuracy}=\frac{\mathrm{TP}+\mathrm{TN}}{\mathrm{TP}+\mathrm{TN}+\mathrm{FN}+\mathrm{FP}}$$

A true positive (TP) is when an event pen is correctly identified (i.e., a true alarm), a true negative (TN) is when a control pen is correctly identified, a false negative (FN) is when an event pen is wrongly identified as a control pen, and a false positive (FP) is when a control pen is wrongly identified as an event pen (i.e., a false alarm). In the current study, the sensitivity is a measure of the proportion of event pens correctly identified by the prediction algorithm, whereas the specificity is a measure of the proportion of control pens correctly identified. The alarm error rate is a measure of the proportion of the alarms (the model predicts that it is an event pen) which are actually false. Accuracy is a measure of the proportion of pens identified correctly by the prediction algorithm, independent of whether they are event or control pens. 

Besides the above measures, the area under the ROC curve (AUC) was also used as a performance measure. The ROC curve was obtained by varying the classification threshold by which the predicted probability was considered an alarm, thereby varying the sensitivity and specificity obtained from the prediction. The AUC was calculated using the ‘auc’ function of the ‘MESS’ library in R [17]. Further, the 95% confidence interval (CI) for the AUC was calculated following the example of Jensen et al. [18] as follows (Equations (10)–(13)):

(10) $$\mathrm{AUC}\pm z_{\alpha /2}\cdot SE_{AUC}$$

where

(11) $$SE_{AUC}=\sqrt{\frac{AUC\cdot \left(1-AUC\right)+\left(N_{1}-1\right)\cdot \left(Q_{1}-AUC^{2}\right)+\left(N_{2}-1\right)\cdot \left(Q_{2}-AUC^{2}\right)}{N_{1}\cdot N_{2}}}$$

(12) $$Q_{1}=\frac{AUC}{2-AUC}$$

(13) $$Q_{2}=\frac{2\cdot AUC^{2}}{1+AUC}$$

and *N*_1_ and *N*_2_ are the numbers of event and control pens, respectively. The performance of the prediction model is said to be better than random guessing if the 95% CI of the AUC does not contain the value 0.5.

#### 2.3.2. Optimisation of Artificial Neural Networks

Event and control pens from batches 1, 2 and 3 were used to optimise and train the ANNs. The predictors included in each ANN for each data source were the standardised summary data of both the raw data and the forecast errors extracted from the DLMs. More precisely, the summary data include the daily minimum, mean, median and maximum values as well as the first and third quantiles. Thus, each ANN included 12 predictors (six on raw data summaries and six on the forecast errors summaries). The response for each ANN was whether the pen was an event pen or a control pen; the value being the same for each pen on all training days included. To get only one prediction per pen, independent on the number of training days included, the maximum predicted probability across the included days was used. As the full day of observation is needed for each day included to calculate the summary data, a potential alarm will first appear at midnight at the end of the particular day.

To avoid bias towards a prediction of a non-event during training, each event pen was only allowed to have one control pen. As most event pens had two control pens available, two training data sets were constructed, each with equal number of event and control pens. ANNs were optimised and trained on each training data set separately. Further, ANNs were optimised for three different combinations of training days included, referred to as three alarm types: (1) alarm ‘untimed’: day-3, day-2 and day-1 (UNTIMED); (2) alarm before event day: day-3 and day-2 (BEFORE); (3) alarm on event day: day-1 (ON). Thus, for each data source, six ANNs were optimised and trained. These three alarm types were tested to investigate whether the precision of the alarm in time would affect the predictive performance of the algorithm.

The optimisation of each ANN was done using n-fold cross-validation where n equals the number of pairs of event and control pens. The training and prediction were iterated n times, and, each time, one of the pairs was not included in the training but instead used for prediction. After the n iterations and based on a 0.5 classification threshold, the sensitivity, specificity and accuracy were calculated (see Section 2.3.1). Each ANN was optimised concerning its activation function(“Rectifier”, “RectifierWithDropout”, “Maxout” or “MaxoutWithDropout”), its number of hidden layers (1 or 2) and its number of nodes in the hidden layers (first layer: 2/3, 1 or 4/3 times the number of predictors; second layer: 2/3, 1 or 4/3 times the number of nodes in the first layer), in total 24 different combinations. Only 1 and 2 hidden layers were tested as it is well-known that a neural network architecture with two hidden layers can learn the same patterns as an architecture with more hidden layers. The combination with the highest accuracy was chosen for each ANN. After the optimisation, the predictive performance of each ANN was evaluated on the training data sets to get a best classification threshold, sensitivity and specificity for each data source and alarm type to be used later in the Bayesian ensemble. The classification threshold was ranging from 0.01 to 1.0 with intervals of 0.01 and the best chosen based on the highest accuracy.

#### 2.3.3. Performance Evaluation of the Artificial Neural Networks

Event and control pens from batch 4 were used to evaluate the performance of the optimised and trained ANNs. As bias was not a concern during the evaluation, each event pen was paired with four or five control pens, depending on availability. A consequence of this was that accuracy could not be used as an evaluation measure. For each data source and alarm type, both ANNs of the two training sets were included in the same prediction. To combine the two ANNs, the average predicted probability was used. This average predicted probability was evaluated with different classification threshold of probabilities ranging from 0.01 to 1.0 with intervals of 0.01. For each classification threshold, the sensitivity, specificity and alarm error rate were calculated, and the threshold evaluated as being most optimal was the one with the highest sum of sensitivity and specificity. Further, a ROC curve was obtained from which the AUC with connected 95% CI was calculated. 

#### 2.3.4. Fixed Probability

Based on a previous risk analysis [7] concerning the three treatments of the study (TAIL, STRAW and SPACE), it was evaluated that the pens would have different fixed probabilities for developing a tail biting event based on the fixed conditions including the three treatments and the week in the study period. To get the fixed probability, all pens from batches 1, 2 and 3 were included. Each pen had one observation per week, and each observation contained information on whether the pen had a tail biting event within the week. These data were included in a simple logistic regression to get the model-estimated probabilities based on the fixed pen data. 

After training the logistic regression on all pens from batches 1, 2 and 3, the model was evaluated on the training data, including the information on the tail biting pens and control pens. This was done to get the best classification thresholds separately for weeks 1–6 and weeks 7–10 for later use in the Bayesian ensemble. The most optimal classification threshold was chosen based on the highest accuracy. For weeks 1–6, classification thresholds ranged from 0.01 to 1.0 with intervals of 0.01, whereas in weeks 7–10 they ranged from 0.001 to 0.01 with intervals of 0.0001.

The logistic regression was also evaluated on event and control pens from batch 4 (the test data) as previously done for the ANNs. The sensitivity and specificity were calculated for different classification thresholds, and the most optimal classification threshold was chosen based on the highest sum of the sensitivity and specificity. This was also done separately for weeks 1-6 and weeks 7–10. At last, a collected sensitivity and specificity for all 10 weeks was calculated. 

#### 2.3.5. Bayesian Ensemble

It was decided to combine the information within each data source and the fixed probability by using Bayes Theorem, also termed a Bayesian ensemble. For our implementation of the Bayesian ensemble, the predicted probability from one data source model was used as the prior probability. The probability is then iteratively updated based on the prediction as well as the sensitivity and specificity of each of the remaining data source models, using Bayes theorem.

The probability of a tail biting event in each pen based on the first model is here termed *P(TB),* and the probability of being a control pen is termed *P(NoTB*). The conditional probabilities of tail biting or no tail biting, given that an alarm was raised or not, by each of the subsequent models are calculated by the sensitivity and specificity of the subsequent model (Equations (14)–(17)):

(14) P(+|TB)=Sensitivity

(15) P(÷|TB)=1−Sensitivity

(16) P(+|NoTB)=1−Specificity

(17) P(÷|NoTB)=Specificity

where + indicates that an alarm was raised by the subsequent model, and ÷ indicates that no alarm was raised by the subsequent model. Using the following formulas (Equations (18) and (19)), the posterior probability of a tail biting event for each pen was calculated:

(18) $$P\left(TB|+\right)=\frac{P\left(+|TB\right)P\left(TB\right)}{P\left(+|TB\right)P\left(TB\right)+P\left(+|NoTB\right)P\left(NoTB\right)}$$

(19) $$P\left(TB|÷\right)=\frac{P\left(÷|TB\right)P\left(TB\right)}{P\left(÷|TB\right)P\left(TB\right)+P\left(÷|NoTB\right)P\left(NoTB\right)}$$

At last, the final posterior probability for each pen was used, again, to calculate sensitivity, specificity and alarm error rate for classification thresholds ranging from 0.01 to 1.0 with intervals of 0.01. The most optimal classification threshold was the one with the highest sum of sensitivity and specificity. Further, the AUC with connected 95% CI was calculated. This Bayesian ensemble was performed for several combinations of the four data sources and the fixed probability. 

### 2.4. Real-Life Application

After testing the performance of the prediction algorithm in identifying pens with a tail biting event from pens without an event, the next step is to test the performance of the prediction algorithm in a real-time setting. This was done by running a number of Bayesian ensemble combinations on all days and pens of batch 4. The combinations chosen were based on the results of the Bayesian ensemble described in Section 2.3.5 and presented in Section 3.4. Thus, this was only performed for the alarm type UNTIMED and included the models and Bayesian ensemble combinations that performed better than random guessing (*n* = 13). To evaluate the performance of each, and as the alarm type UNTIMED was used, the tail biting event predictions were clustered in 3 days prior to the events, meaning that if an alarm occurred on any of the 3 days prior to an event, this was counted as one true positive. If an alarm did not occur on any of the 3 days prior to the event, this was counted as one false negative. If an alarm occurred on any day that was not 3 days prior to an event, this was counted as a false positive, and if no alarm occurred on any day that was not 3 days prior to an event, this was counted as a true negative. From this, sensitivity, specificity and alarm error rate were calculated for classification thresholds ranging from 0.01 to 1.0 with 0.01 intervals. The best classification threshold was the one with the highest sum of sensitivity and specificity. Further, the AUC with connected 95% CI was calculated.

## 3. Results

### 3.1. Model Parameters

**Figure 2:**
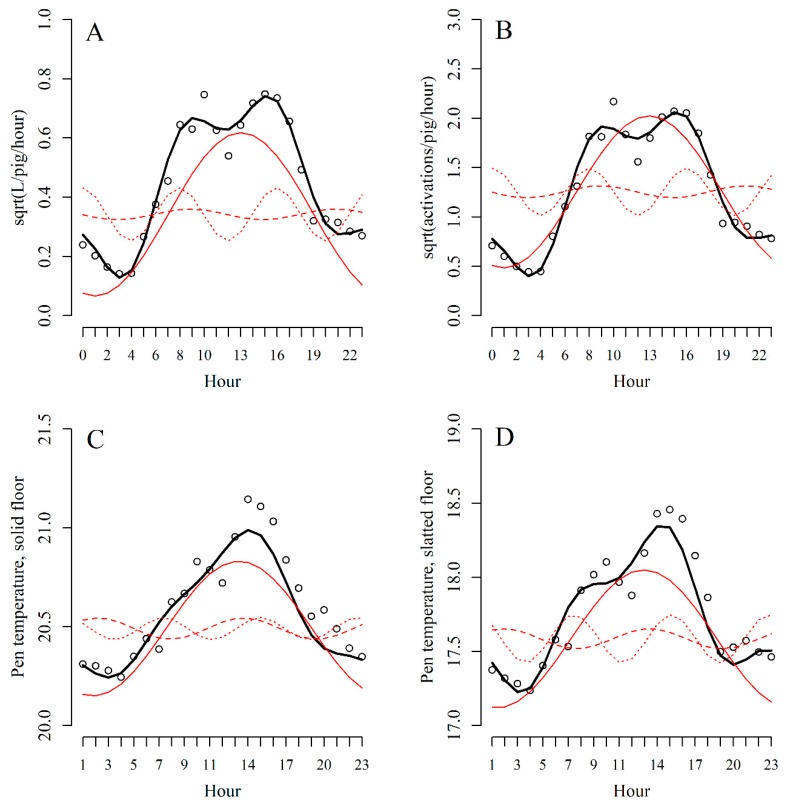
Diurnal pattern of (**A**) water flow, (**B**) activation frequency, (**C**) pen temperature above the solid floor and (**D**) pen temperature above the slatted floor, averaged across all days of the study. Circles: mean observation of the raw data for each hour. Thin red lines: the model-estimated three harmonic waves with 24-h (—), 12-h (- - -) and 8-h (····) cycles. Thick black line: the model-estimated sum of the three harmonic waves. No recordings were available on pen temperature for the first hour of the day (hour 0).

The observational variances for the four DLMs were optimised with the following percentages of the mean values of the four data sources: water flow: 7.50%; activation frequency: 16.00%; pen temperature above the solid floor: 0.40%; pen temperature above the slatted floor: 0.65%. The following discount factors were used for the four DLMs: water flow: 0.98; activation frequency: 0.97; pen temperature above the solid floor: 0.88; pen temperature above the slatted floor: 0.88. Model estimates of the initial mean and hourly linear trend for each data source as well as model estimates of *A* and *c* for each of the three waves for each data source can be seen in the Supplementary Material (Tables S1 and S2). The model-estimated diurnal pattern for each data source is shown in Figure 2.

### 3.2. Performance of The Artificial Neural Networks

The optimisation combination chosen for each ANN as well as the predictive performance of each ANN when evaluated on the training data sets can be seen in the Supplementary Material (Table S3). The predictive performance of the ANNs for each data source and alarm type when evaluated on the test data set can be seen in Table 1. Only the alarm type UNTIMED performed better than random guessing and only for the data sources water flow and pen temperature above the solid floor. For water flow, 82% of the event pens (*n* = 9) were correctly identified, whereas this only applied to 58% of the control pens (*n* = 29), resulting in many false alarms and a high alarm error rate with 70% of the alarms being false. For pen temperature above the solid floor, 67% of the event pens (*n* = 8) and 72% of the control pens (*n* = 39) were correctly identified with 65% of the alarms being false. Thus, there is a definite potential for improvement in the predictive performance, and it does not seem enough to include only one data source in the prediction of pens with tail biting events.

### 3.3. Performance of the Fixed Probability Model

The probabilities estimated from the fixed probability model can be seen in Table 2, and its predictive performance when evaluated on the training data sets can be seen in the Supplementary Material (Table S4). The values of Table 2 clearly show, as expected, that undocked tails, no straw provision and low space allowance additively increase the probability of a tail biting event. Further, the probability seems to decrease with time with a large drop from week 6 to 7. From week 1 to 6, the best classification threshold was 0.13, whereas it was 0.0068 from week 7 to 10. When using these two classification thresholds for the different weeks of the study, a sensitivity of 0.818 and a specificity of 0.800 were obtained, meaning that 82% (*n* = 10) of the event pens and 80% of the control pens (*n* = 43) were correctly identified and ‘only’ 52% of the raised alarms were false. Thus, from these results, using the general and fixed characteristics of the pen made a better prediction than any of the dynamic data sources. However, it has to be remembered that if only using the fixed probability, the model will predict an event in the particular pen for each day of the particular week and not only on the day of the event. Thus, in practise, the fixed probability model will not be able to stand alone. Instead, it can be used to update the prediction of the dynamic data sources as done in the current study with the Bayesian ensemble.

### 3.4. Performance after the Bayesian Emsemble

The classification thresholds, sensitivities and specificities used for the Bayesian ensemble for each model included were the average of the values obtained from the performance evaluation on the training data sets (see Tables S3 and S4).

**Figure 3:**
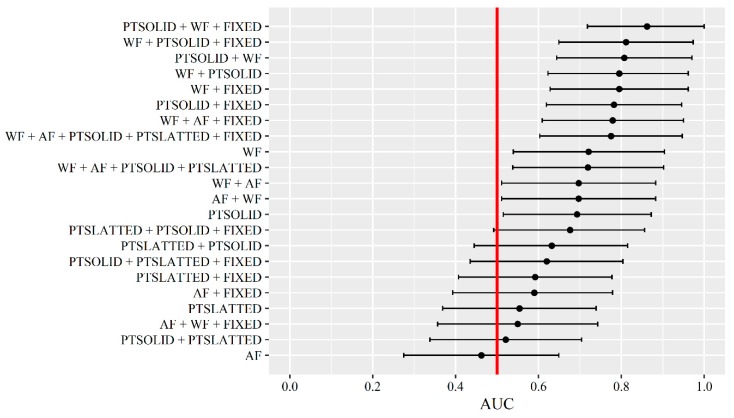
AUC (area under the ROC curve) with 95% confidence intervals (CI) for each Bayesian ensemble of models on water flow (WF), activation frequency (AF), pen temperature above the solid floor (PTSOLID), pen temperature above the slatted floor (PTSLATTED) and the fixed probability (FIXED) for the alarm type UNTIMED (day-1, day-2 and day-3). If the CI does not overlap with the red vertical line, the final updated model has a predictive performance better than random guessing.

The predictive performance of each Bayesian ensemble combination of the data source and fixed probability models including the sensitivity, specificity and alarm error rate can be found in the supplemental material for alarm type UNTIMED (Table S5), alarm type BEFORE (Table S6) and alarm type ON (Table S7). The AUC with 95% CI for each Bayesian ensemble combination for the alarm type UNTIMED (day-3, day-2 and day-1 included) is shown in Figure 3. 

For the alarm type BEFORE, only the Bayesian ensemble combination including the models on pen temperature above the solid floor and the fixed probability performed better than random guessing, although with an AUC just below 0.70. Otherwise, no Bayesian ensemble combinations within the alarm types BEFORE and ON performed better than random guessing. 

For the alarm type UNTIMED, 13 Bayesian ensemble combinations performed better than random guessing with three of the combinations having an AUC above 0.80 (see Figure 3). The two best combinations included the models on water flow, pen temperature above the solid floor and the fixed probability, and it seemed to be important which of the data sources were used first in the Bayesian ensemble. Excluding the fixed probability from the ensemble still resulted in AUCs close to 0.80. On the other hand, neither activation frequency nor pen temperature above the slatted floor seemed important for the identification of pens with tail biting events.

Overall, water flow and pen temperature above the solid floor seem to be the data sources important for the identification of pens with a tail biting event, and the fixed probability of the pen seems able to improve the performance of the identification. Further, to achieve a good enough performance of the identification, it seems necessary to compromise on the timely precision of the identification, as it was necessary to include all 3 days prior to the event.

### 3.5. Real-Life Application

**Figure 4:**
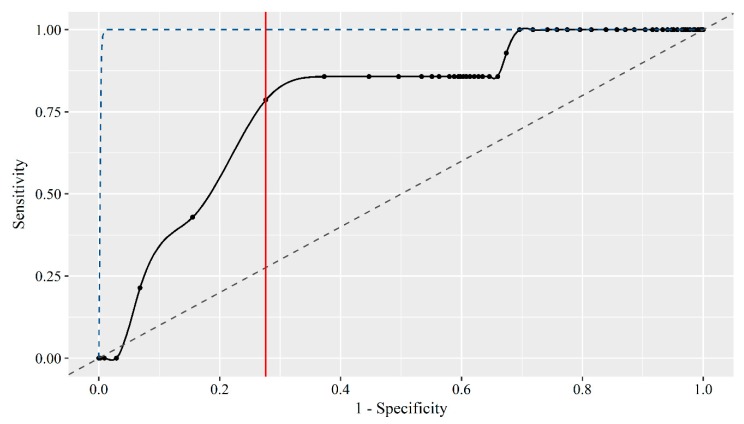
Receiver operating characteristic (ROC) curve for the best performing prediction algorithm of tail biting events in a real-life setting being a Bayesian ensemble combination of all four data sources within the alarm type UNTIMED (day-1, day-2 and day-3). The black solid line and points represent the specificity and sensitivity at different classification thresholds. The grey dashed line represents a prediction algorithm equal to random guessing, whereas the blue dashed line represents a prediction algorithm with an ideal performance. The red solid vertical line represents the best classification threshold being the one with the highest sum of sensitivity and specificity.

Performance results of the 13 different models and Bayesian ensemble combinations tested in a real-life setting can be seen in Table 3. Further, the performance results are presented in Table 4 as the number of event days with an alarm for both tail biting, fouling and diarrhoea events. Table 4 also presents the number of no-event days (no tail biting event), day0 days and day+1 days with an alarm (all false positive). The Bayesian ensemble combination with the highest AUC in the prediction of tail biting events in a real-life setting was the one including all four data sources (AUC = 0.769). Although this combination ‘only’ predicted 11 of the 14 tail biting events, it did so while giving alarms on ‘only’ 28% of the no-event days (*n* = 553). Further, it predicted 25 of the 35 fouling events and 13 of the 16 diarrhoea events. If solely prioritising to predict the tail biting events, alarms would also be given on 46–64% of the no-event days. The receiver operating characteristic (ROC) curve of the best Bayesian ensemble combination as presented above is shown in Figure 4 and shows that the prediction is better than random guessing but, also, that there is great room for improvement.

## 4. Discussion

The purpose of the current study was to develop a prediction algorithm for tail biting events in finisher pigs based on already available sensor data. To develop such a prediction algorithm took several steps and resulted in a real-life setting algorithm with an AUC > 0.75, although with many false alarms. Thus, both pigs’ water usage and pen temperature seem to have predictive value for tail biting. In the following, the results will be discussed in connection to the data sources included, the timing of the alarms, the number of false alarms and possible improvements to the prediction algorithm.

### 4.1. Which Data Sources to Include

Only water flow and pen temperature above the solid floor were better than random guessing at predicting tail biting events when considered on their own, and this was also the case in a real-life setting. Water flow seemed to be the most sensitive data source with all tail biting events predicted. However, both water flow and pen temperature above the solid floor resulted in around 50% of the no-event days with false alarms, which in this study is around 1000 alarms that the farmer should react on to be able to prevent 14 tail biting events. Using the Bayesian ensemble strategy has previously been proved successful in improving the predictive performance of fouling events [19]. Therefore, it was chosen also to try this strategy in the current study. Combining the data sources using the Bayesian ensemble strategy seemed to lower the number of false alarms with the compromise of lowering the number of tail biting events predicted. The Bayesian ensemble combinations with the highest performance in identifying tail biting pens from control pens included water flow and pen temperature above the solid floor, while in a real-life setting these combinations performed the worst when considering the number of false alarms. Further, when including the fixed probability model in any combination, the performance in a real-life setting only worsened, whereas the opposite was found when trying to identify tail biting event pens from control pens. This underpins the importance of not concluding too firmly on the predictive performance of a data source or ensemble of data sources before investigating this in a real-life setting as well as including data sources where such an investigation is possible, e.g., continuously measured sensor data.

Unexpectedly, the activation frequency seemed able to lower the number of false alarms when combined with water flow in a real-life setting. With this combination, 12 of the 14 tail biting events were predicted, and ‘only’ 31% of the no-event days were false alarms, lowering the number of false alarms with around 400. The fact that this combination performs well is in accordance with previous investigations, e.g., Jensen et al. [18] who showed that data on drinking amount and drinking frequency contain mutually independent information value with respect to early detection of undesired events in finisher pigs. Also Dominiak et al. [20] found AUCs > 0.80 when using similar data sources to predict tail biting events in both weaner and finisher pigs using both internal (as in the current study) and external evaluation. The higher predictive performance found by Dominiak et al. [20] could be due to differences in the gold standard used. In the current study, the gold standard was observed in detail within the pen by looking at each individual tail three times per week combined with daily observations from outside the pen. In the study by Dominiak et al. [20], only the latter method was used. The threshold for a tail biting event in the current study may have been less severe and may have been observed at an earlier stage where the possible behavioural changes could be less pronounced. Further, Dominiak et al. [20] also included the day of the event in their time window. Thus, the algorithm used by Dominiak et al. [20] may have been identifying events already in the outbreak stage with severe tail damage, whereas the current algorithm was developed to predict these events prior to the serious tail damage. Further, the two investigations used different approaches in developing the prediction algorithms.

The Bayesian ensemble combination with the highest AUC and the least false alarms included all four data sources. However, when compared to the combination including only water flow and activation frequency, the false alarms were 3% less (69 less false alarms), and one less tail biting event was predicted. Thus, it is a question of costs and benefits and the interest of the farmer when asking how many data sources should be included. In this case, each added data source will add the cost of another sensor and/or system to handle the sensor data for each pen at the herd. An additional consideration to remember is whether the farmer is most interested in all tail biting events being predicted and cares less about the false alarms, or whether he will rather miss some tail biting events and have fewer false alarms. Naturally, this also depends on the cost of each tail biting event and of each false alarm, which again depends on how the farmer reacts to the alarms. Does he merely use the alarms as a tool to pay more attention to the alarmed pens, or does he actively try to prevent tail biting at each alarm? In the latter case, a reduction in the number of false alarms should be prioritised although a lack of focus on the sensitivity of the prediction will make a prediction algorithm for tail biting events dispensable. Another approach would be to provide the farmer with the probability of a tail biting event for each pen on each day based on the prediction algorithm and then make him decide the threshold for the alarms based on the time he has available for handling such alarms. Thus, in periods with more time available, the threshold would be lower, and the farmer would prevent more tail biting events, but he will probably also get more false alarms. In periods with less time available, the threshold will be higher, the farmer will prevent less tail biting events, but he will also have less false alarms. This will give the farmer the flexibility to make the alarm system work within his specific herd.

### 4.2. Timing of Alarms

Previously developed prediction algorithms for finisher pigs, focussing on prediction of tail biting, fouling and diarrhoea events, investigated time windows including the day of the event and sometimes the day after the event [18,20,21]. This means that the farmer can possibly receive an alarm after the event, probably while the event is still ongoing but at a later stage of development. As the intention with this study was that the farmer should get an alarm prior to serious tail damage, alarm types investigating these days relative to the event were not included in the current study.

The optimal prediction algorithm will produce an alarm one or a few days before the event, making it possible for the farmer to prevent the event. However, when considering the high number of false alarms, it would perhaps make more sense to the farmer to receive the alarm on the day of the event to be able to observe whether the event has actually happened, although still at an early stage. Both scenarios were investigated in the current study in the form of the BEFORE and ON alarm types. However, none of the prediction algorithms investigated within each of the two alarm types showed usable predictive performances when trying to identify a tail biting event pen from a control pen. Thus, they were not tested in a real-life setting. 

Instead, it seemed necessary to compromise in the timing of the alarms to get usable predictive performances. This means that with the prediction algorithms tested in the real-life setting in the current study, the farmer will not know whether the alarm occurs 1 or 2 days before the event or on the day of the event. Thus, in this case, the farmer would benefit the most from using the alarms as an indicator of which pens to be more aware of during the days to come.

### 4.3. The Meaning of False Alarms

The greatest challenge when developing prediction algorithms for event-type behaviour is to reduce the great number of false alarms and thus the high alarm error rate [22]. This challenge appears because the event that is predicted occurs infrequently compared to the number of periods (here days) without the event. However, the false alarms may still appear for a biological reason. Perhaps the water usage and pen temperature in pens of finisher pigs change for other reasons than merely tail biting. 

An investigation using data from the same experiment as the current one found that pen temperature above the solid floor decreased prior to pen fouling, probably as a consequence of more and more pigs moving from resting in the solid floor area to other areas of the pen [11]. Further, yet unpublished results from the same experiment show a higher water flow during the two diurnal peaks in pens with a diarrhoea event compared to non-event pens the last 5 days prior to the event. Likewise, a higher activation frequency was found in the diarrhoea event pens without straw. Thus, both water usage and pen temperature seem related to other undesirable events in finisher pigs. This hypothesis fits well with the results of the current investigation, as a part of the false alarms were true alarms if considered predictions of fouling and diarrhoea events even though the algorithm was not developed to predict these events. Further, the percentage of false alarms explained by fouling and diarrhoea events would probably be even greater if the day of the events was also included, as these could be for longer periods until the events were controlled properly. 

Previous investigations into the prediction of undesirable events in finisher pigs have also looked at water usage and pen temperature as predictors of pen fouling and diarrhoea [18,20,23]. Jensen et al. [18] tried to indiscriminately predict pen fouling and diarrhoea events using a multivariate dynamic linear model including predictors on water usage, pen temperature, humidity and feed usage. They found that both water usage and pen temperature had predictive value of the two events with AUCs > 0.80 for water usage and AUCs > 0.75 for pen temperature, whereas the other predictors did not. Dominiak et al. [23] also tried to indiscriminately predict events of pen fouling and diarrhoea. They only included water usage as a predictor in a spatial dynamic linear model, but they also found AUCs > 0.80. When solely trying to predict diarrhoea events using the same model on a different finisher herd, the AUC dropped to just above 0.70 [20]. 

Overall, the water usage and pen temperature in pens of finisher pigs seem to depend on more than the development of tail biting into tail damage, and changes in these parameters may be a result of a more general stress response. Thus, a false alarm may still indicate that a pen needs more attention. 

### 4.4. How to Improve the Prediction Algorithm

If a prediction algorithm, as the one developed in the current study, should be of use to the farmer, the number of false alarms should be reduced or the reason for them specified. As has been shown in both this and other studies, deviations in temperature and drinking behaviour from the expected patterns can raise alarms of multiple undesired events. To our knowledge, however, no one has yet investigated to what extent, if any, specific patterns of these deviations might be able to provide more specific alarms. In this study, we trained ANNs to specifically distinguish pens with tail biting from pens with no tail biting based on deviations from the expected patterns, yet these ANNs would very consistently also detect cases of diarrhoea and pen fouling. An alternative approach could be to train one model to first distinguish between normal/healthy pens and pens with any undesired event. A secondary model could then be trained and used to distinguish between different types of undesired events, such as diarrhoea, tail biting and pen fouling. This possibility will be investigated in future studies. 

Additionally, a reduction in false alarms and more event-specific alarms could potentially be achieved by including more event-specific predictors. Piglets tuck their tail between the legs after having been tail docked [24], and thus it has been hypothesised for a long time that pigs may also change their tail posture as a reaction to on-going tail biting. Recently conducted investigations confirm that the tail posture of pigs does change prior to tail biting events towards more pigs in a pen with a lowered tail, and thus lowered tails seem to be a reaction to on-going tail biting in both weaner and finisher pigs [25,26]. Therefore, tail posture may be an event-specific predictor for tail biting, making it possible for the algorithm to distinguish between tail biting and other undesirable events. Future research should focus on developing a method for automatic recording of tail posture of pigs to be able to investigate tail posture as a predictor of tail biting events in a real-life setting. Such research work has already been initialised by D’Eath et al. [27]. Both activity level and object manipulation have also been shown to change prior to tail biting events [10] and thus may also be valuable predictors in the algorithm when it may be possible to record these automatically in the future by the use of image analysis and/or accelerometers. 

For pen fouling, a more event-specific predictor could be changes in the lying pattern of the pigs. Larsen et al. [11] saw fewer and fewer pigs lying on the solid floor and more and more pigs lying on the slatted floor the last 3 days prior to an event of pen fouling, although only in pens not provided with straw on the solid floor. Also, Jensen et al. [19] investigated the predictive value of the location of finisher pigs in the pen in relation to pen fouling and found AUCs > 0.70. Further, an automatic recording method for pig location in the pen is currently under development using convolutional neural networks. For diarrhoea, a more event-specific predictor could be related to the performance of sickness behaviour including a lowered activity level. However, to the knowledge of the authors, this has not yet been investigated, and changes in activity level may be a too general predictor as well as it could be related to many other changes in the environment or other events including tail biting [10]. However, the changes seen in activity level may be different for the different events.

Thus, it seems possible to find more event-specific predictors, but automatic recording methods for these predictors are still lacking and should be the focus for future research.

## 5. Conclusions

A prediction algorithm for the prediction of tail biting events in a real-life setting was developed with an AUC > 0.75 and ‘only’ around 30% of the non-event days having false alarms. The algorithm with the highest AUC included all four data sources, but the performance of the prediction was only slightly better than the algorithm including predictors on water usage only. Due to the high number of false alarms, it was suggested that farmers use the alarms produced by the algorithm to pay greater attention to the alarmed pens and not try to actively prevent tail biting at every alarm. The algorithm also raised alarms prior to events of pen fouling and diarrhoea. Thus, future research should focus on investigating more event-specific predictors, such as tail posture for events of tail biting, and on developing automatic recording methods for these predictors.

## Figures and Tables

**Table 1:** Predictive performance of the artificial neural networks trained and optimised for each data source and alarm type.

Alarm Type ^A^	Data Source	No. of Event Pens	No. of Control Pens	AUC ^B^	95% CI	Best Threshold ^C^	Sensitivity ^D^	Specificity ^D^	Alarm Error Rate ^D^
UNTIMED	Water flow	11	50	0.721	0.539–0.904	0.57	0.818	0.580	0.700
	Activation frequency	11	50	0.462	0.275–0.649	0.90	0.182	0.920	0.666
	Pen temperature, solid	12	54	0.693	0.515–0.872	0.77	0.667	0.722	0.652
	Pen temperature, slatted	12	54	0.554	0.369–0.739	0.45	0.917	0.241	0.788
BEFORE	Water flow	11	50	0.525	0.334–0.717	0.64	0.455	0.700	0.750
	Activation frequency	11	50	0.438	0.255–0.622	0.64	0.364	0.700	0.636
	Pen temperature, solid	12	54	0.581	0.396–0.766	0.68	0.750	0.519	0.743
	Pen temperature, slatted	12	54	0.444	0.268–0.620	0.83	0.167	0.907	0.714
ON	Water flow	12	54	0.500	0.318–0.682	0.27	1.000	0.167	0.789
	Activation frequency	12	54	0.510	0.327–0.692	0.85	0.333	0.833	0.692
	Pen temperature, solid	12	54	0.568	0.383–0.753	0.26	0.917	0.264	0.780
	Pen temperature, slatted	12	53	0.471	0.291–0.650	0.62	1.000	0.208	0.778

^A^ The number of days relative to a tail biting event included in the training data representing getting an alarm UNTIMED prior to the event (Day-1, -2, -3), 1 or 2 days BEFORE the event (Day-2, -3) and ON the day of the event (Day-1). ^B^ The area under the receiver operating characteristic curve. ^C^ The classification threshold with the highest sum of sensitivity and specificity. ^D^ The performance measures achieved by using the best threshold.

**Table 2:** The probability of a tail biting event for each combination of the three treatments (TAIL, STRAW and SPACE) in each week of the study period.

TAIL	STRAW	SPACE	Week									
			1	2	3	4	5	6	7	8	9	10
Docked	Yes	Low	3.61	2.55	3.98	2.55	3.24	1.91	0.24	0.50	1.03	0.76
		High	6.72	4.80	7.40	4.80	6.06	3.62	0.47	0.95	1.97	1.45
	No	Low	9.38	6.76	10.29	6.76	8.48	5.11	0.67	1.36	2.80	2.07
		High	16.61	12.25	18.09	12.25	15.15	9.40	1.28	2.59	5.26	3.91
Undocked	Yes	Low	10.09	7.29	11.07	7.29	9.13	5.52	0.73	1.47	3.03	2.24
		High	17.77	13.14	19.33	13.14	16.22	10.12	1.39	2.80	5.68	4.23
	No	Low	23.68	17.85	25.60	17.85	21.75	13.91	1.98	3.97	7.95	5.96
		High	37.40	29.50	39.85	29.50	34.87	23.73	3.75	7.38	14.27	10.88

**Table 3:** Predictive performance of chosen Bayesian ensemble combinations tested in a real-life setting using the alarm type UNTIMED (day-1, day-2 and day-3 included; WF: water flow, AF: activation frequency, PTSOLID: pen temperature above the solid floor, PTSLATTED: pen temperature above the slatted floor, FIXED: fixed probability). All single models and combinations evaluated 14 tail biting events.

Bayesian Ensemble Combination	No. of No-Event Days	AUC ^A^	95% CI	Best Threshold ^B^	Sensitivity ^C^	Specificity ^C^	Alarm Error Rate ^C^
WF	2052	0.756	0.608–0.904	0.54	1.000	0.536	0.986
PTSOLID	2061	0.686	0.530–0.841	0.99	0.857	0.475	0.989
WF + PTSOLID	2031	0.762	0.615–0.909	0.67	1.000	0.535	0.985
WF + FIXED	2052	0.726	0.574–0.878	0.99	0.857	0.559	0.987
WF + PTSOLID + FIXED	2031	0.678	0.522–0.834	0.59	0.929	0.440	0.989
WF + AF	2052	0.761	0.614–0.908	0.66	0.857	0.692	0.981
AF + WF	2052	0.722	0.569–0.874	0.48	0.929	0.503	0.987
WF + AF + FIXED	2052	0.725	0.573–0.877	0.58	0.786	0.637	0.985
PTSOLID + WF	2031	0.726	0.574–0.878	0.99	0.857	0.559	0.987
PTSOLID + FIXED	2061	0.653	0.496–0.810	0.99	0.786	0.470	0.990
PTSOLID + WF + FIXED	2031	0.659	0.502–0.816	0.99	0.714	0.549	0.989
WF + AF + PTSOLID + PTSLATTED	2031	0.769	0.623–0.915	0.93	0.786	0.724	0.981
WF + AF + PTSOLID + PTSLATTED + FIXED	2031	0.726	0.575–0.878	0.90	0.786	0.635	0.985

^A^ The area under the receiver operating characteristic curve. ^B^ The classification threshold with the highest sum of sensitivity and specificity. ^C^ The performance measures achieved by using the best threshold.

**Table 4:** Number of no-event days, prior-event days (Day-3:Day-1), on-event days (Day 0) and post-event days (Day+1) identified as having an alarm out of the total number of each day type for tail biting, fouling and diarrhoea events. The alarm type UNTIMED was used, including the models and Bayesian ensemble combination performing better than random guessing when trying to identify pens with a tail biting event from control pens (WF: water flow, AF: activation frequency, PTSOLID: pen temperature above the solid floor, PTSLATTED: pen temperature above the slatted floor, FIXED: fixed probability).

Bayesian Update Combination	Tail Biting					Fouling	Diarrhoea
	No-Event Day ^A^		Day-3:Day-1 ^A^	Day 0 ^A^	Day + 1 ^A^	Day-3:Day-1 ^A^	Day-3:Day-1 ^A^
WF	937/2023	(46%)	14/14	8/14	8/15	25/35	13/16
PTSOLID	1065/2033	(52%)	12/14	8/14	8/14	22/35	14/16
WF + PTSOLID	1278/2003	(64%)	14/14	8/14	8/14	25/35	13/16
WF + FIXED	1150/2023	(57%)	13/14	9/14	12/15	24/35	12/16
WF + PTSOLID + FIXED	1117/2003	(56%)	13/14	8/14	12/14	24/35	13/16
WF + AF	622/2023	(31%)	12/14	6/14	4/15	25/35	10/16
AF + WF	1007/2023	(50%)	13/14	6/14	6/15	25/35	13/16
WF + AF + FIXED	734/2023	(36%)	11/14	6/14	5/15	17/35	11/16
PTSOLID + WF	883/2003	(44%)	12/14	7/14	9/14	25/35	13/16
PTSOLID + FIXED	1078/2033	(53%)	11/14	7/14	8/14	22/35	13/16
PTSOLID + WF + FIXED	901/2003	(45%)	10/14	7/14	7/14	17/35	11/16
WF + AF + PTSOLID + PTSLATTED	553/2003	(28%)	11/14	4/14	3/14	25/35	13/16
WF + AF + PTSOLID + PTSLATTED + FIXED	729/2003	(36%)	11/14	6/8	6/8	17/35	11/16

^A^ Results obtained when using the best classification threshold as presented in Table 3.

## Supplementary Materials

The following is available online at https://www.mdpi.com/2076-2615/9/7/458/s1: Table S1: Model estimates of initial mean and linear trend, Table S2: Model estimates of harmonic wave parameters, Table S3: ANN optimisation results, Table S4: Predictive performance of FIXED model, Table S5: Predictive performance of alarm type UNTIMED; Table S6: Predictive performance of alarm type BEFORE, Table S7: Predictive performance of alarm type ON.
