# Supplementary material for: Prediction of Tail Biting Events in Finisher Pigs from Automatically Recorded Sensor Data
Source: Animals (Basel). 2019 Jul 19;9(7):458. doi: 10.3390/ani9070458 (PMC6681100; doi:10.3390/ani9070458)
Supplement: Supplementary file 1 [file animals-09-00458-s001.pdf]

Table S1. Model estimates of initial mean and hourly growth trend for each of the four data sources. Estimates on water flow and activation frequency are from square root transformed data.

Data source	Initial mean	Hourly growth trend
Water flow (L/pig)	0.34	0.000132
Activation frequency (no./pig)	1.25	0.000043
Pen temperature, solid floor (°C)	21.30	-0.000417
Pen temperature, slatted floor (°C)	18.00	-0.001940

Table S2. Model estimates of amplitude (*A*) and phase shift (*c*) for each of the three harmonic waves (wave 1: 24 h cycle; wave 2: 12 h cycle; wave 3: 8 h cycle) for each of the four data sources as well as the period (*b*) provided to each of the three waves for each data source. Estimates on water flow and activation frequency are from square root transformed data.

Data source	*A*	*b*	*c*
Water flow			
Wave 1	0.276	$1 \cdot (2 \cdot \pi)/24$	-1.84
Wave 2	0.018	$2 \cdot (2 \cdot \pi)/24$	-3.06
Wave 3	0.090	$3 \cdot (2 \cdot \pi)/24$	1.62
Activation frequency			
Wave 1	0.770	$1 \cdot (2 \cdot \pi)/24$	-1.82
Wave 2	0.060	$2 \cdot (2 \cdot \pi)/24$	-3.07
Wave 3	0.240	$3 \cdot (2 \cdot \pi)/24$	1.57
Pen temperature, solid floor			
Wave 1	0.341	$1 \cdot (2 \cdot \pi)/24$	-2.06
Wave 2	0.053	$2 \cdot (2 \cdot \pi)/24$	0.39
Wave 3	0.056	$3 \cdot (2 \cdot \pi)/24$	1.84
Pen temperature, slatted floor			
Wave 1	0.465	$1 \cdot (2 \cdot \pi)/24$	-1.97
Wave 2	0.067	$2 \cdot (2 \cdot \pi)/24$	0.53
Wave 3	0.163	$3 \cdot (2 \cdot \pi)/24$	1.73

Table S3. Optimisation results for each data source, investigated by n-fold cross validation (n = number of pairs of event and control pens) with different number of days included in the training data. Each ANN was optimised according to its activation function, number of layers and number of nodes in the hidden layers.

Data source	Training days	Training dataset	No. of pairs	Activation function[a]	No. of hidden layers[b]	No. of nodes in hidden layers[c]	Best threshold[d]	Accuracy[e]	Sensitivity[e]	Specificity[e]
Water flow	-1	1	37	Rectifier	1	(3/3)* predictors	0.46	0.649	0.649	0.649
		2	30	RectifierWithDropout	1	(2/3)* predictors	0.47	0.717	0.767	0.667
	-2:-3	1	33	MaxoutWithDropout	2	(4/3)* predictors	0.45	0.667	0.788	0.545
		2	27	MaxoutWithDropout	2	(4/3)* predictors	0.45	0.630	0.852	0.407
	-1:-3	1	32	MaxoutWithDropout	2	(4/3)* predictors	0.58	0.609	0.625	0.594
		2	25	MaxoutWithDropout	2	(2/3)* predictors	0.56	0.660	0.800	0.520
Activation frequency	-1	1	37	Rectifier	2	(3/3)* predictors	0.41	0.581	0.514	0.649
		2	30	MaxoutWithDropout	1	(2/3)* predictors	0.37	0.767	0.833	0.700
	-2:-3	1	33	RectifierWithDropout	1	(2/3)* predictors	0.61	0.652	0.576	0.727
		2	27	RectifierWithDropout	2	(3/3)* predictors	0.59	0.685	0.593	0.778
	-1:-3	1	32	Rectifier	1	(3/3)* predictors	0.51	0.625	0.938	0.312
		2	25	MaxoutWithDropout	1	(3/3)* predictors	0.62	0.700	0.760	0.640
Pen temperature, solid floor	-1	1	32	Maxout	1	(2/3)* predictors	0.48	0.672	0.656	0.688
		2	25	MaxoutWithDropout	1	(2/3)* predictors	0.39	0.660	0.920	0.400
	-2:-3	1	29	Rectifier	1	(2/3)* predictors	0.78	0.655	0.448	0.862
		2	23	Rectifier	2	(2/3)* predictors	0.42	0.630	0.826	0.435
	-1:-3	1	27	Rectifier	2	(3/3)* predictors	0.47	0.648	0.815	0.481
		2	21	Rectifier	2	(2/3)* predictors	0.83	0.595	0.333	0.857
Pen temperature, slatted	-1	1	34	Rectifier	2	(4/3)* predictors	0.49	0.647	0.441	0.853
		2	27	Rectifier	2	(2/3)* predictors	0.52	0.685	0.519	0.852
	-2:-3	1	29	MaxoutWithDropout	1	(2/3)* predictors	0.71	0.672	0.517	0.828
		2	23	Rectifier	1	(2/3)* predictors	0.58	0.717	0.739	0.696
	-1:-3	1	29	Rectifier	2	(2/3)* predictors	0.76	0.672	0.448	0.897
		2	22	Rectifier	2	(2/3)* predictors	0.56	0.659	0.455	0.864

[a] Choice between "Rectifier", "RectifierWithDropout", "Maxout" and "MaxoutWithDropout".

[b] Choice between 1 and 2 hidden layers.

[c] Choice between (2/3)*predictors, (3/3)* predictors and (4/3)*predictors in layer 1 and (2/3)*layer 1 nodes, (3/3)* layer 1 nodes and (4/3)* layer 1 nodes in layer 2

[d] The best classification threshold yielding the highest accuracy (investigated from 0.01 to 1.00 with 0.01 intervals).

[e] The performance measures achieved by using the best classification threshold.

Table S4. Predictive performance and best classification threshold for the fixed probability model when investigated on the two training datasets and separately for week 1-6 and week 7-10.

Weeks	Training dataset	No. of pairs	Best threshold[a]	Accuracy[b]	Sensitivity[b]	Specificity[b]
1 - 6	1	32	0.17	0.594	0.531	0.656
	2	27	0.17	0.667	0.481	0.852
7 - 10	1	5	0.026	0.700	0.600	0.800
	2	3	0.014	0.667	0.667	0.667

[a] The best classification threshold yielding the highest accuracy (week 1-6: 0.01 to 1.00 with 0.01 intervals; week 7-10: 0.001 to 0.1 with 0.0001 intervals).

[b] The performance measures achieved by using the best classification threshold.

Table S5. Predictive performance for the alarm type UNTIMED (training day -1, -2 and -3 included) for each of the four data source models (WF: water flow, AF: activation frequency, PTSOLID: pen temperature above the solid floor, PTSLATTED: pen temperature above the slatted floor) and when combined with each other and the model on fixed probabilities (FIXED) through the Bayesian ensemble strategy.

Bayesian ensemble combination	No. of event cases	No. of control cases	AUC	95% CI	Best threshold[a]	Sensitivity[b]	Specificity[b]	Alarm error rate[b]
WF	11	50	0.721	0.539-0.904	0.57	0.818	0.580	0.700
WF + FIXED	11	50	0.795	0.628-0.961	0.47	0.909	0.580	0.677
AF	11	50	0.462	0.275-0.649	0.90	0.182	0.920	0.667
AF + FIXED	11	50	0.590	0.393-0.779	0.53	0.727	0.520	0.750
WF + AF	11	50	0.697	0.511-0.883	0.62	0.727	0.640	0.692
WF + AF + FIXED	11	50	0.779	0.609-0.950	0.59	0.818	0.740	0.590
AF + WF	11	50	0.485	0.297-0.674	0.73	0.455	0.760	0.706
AF + WF + FIXED	11	50	0.550	0.357-0.743	0.85	0.364	0.900	0.556
PTSOLID	12	54	0.693	0.515-0.872	0.77	0.667	0.722	0.652
PTSOLID + FIXED	12	54	0.782	0.619-0.945	0.69	0.917	0.667	0.621
PTSLATTED	12	54	0.554	0.369-0.739	0.45	0.917	0.241	0.788
PTSLATTED + FIXED	12	54	0.592	0.407-0.777	0.45	0.583	0.704	0.696
PTSOLID + PTSLATTED	12	54	0.521	0.338-0.704	0.46	0.917	0.259	0.784
PTSOLID + PTSLATTED + FIXED	12	54	0.620	0.435-0.804	0.64	0.583	0.685	0.708
PTSLATTED + PTSOLID	12	54	0.632	0.445-0.815	0.27	0.917	0.370	0.756
PTSLATTED + PTSOLID + FIXED	12	54	0.676	0.492-0.856	0.19	1.000	0.333	0.750
WF + PTSOLID	11	50	0.795	0.623-0.961	0.58	1.000	0.580	0.656
WF + PTSOLID + FIXED	11	50	0.811	0.649-0.973	0.48	0.923	0.623	0.657
PTSOLID + WF	11	50	0.807	0.644-0.970	0.67	0.909	0.600	0.667
PTSOLID + WF + FIXED	11	50	0.862	0.718-1.000	0.69	0.909	0.760	0.545
WF + AF + PTSOLID + PTSLATTED	11	50	0.719	0.538-0.902	0.65	0.727	0.720	0.636
WF + AF + PTSOLID + PTSLATTED + FIXED	11	50	0.775	0.603-0.947	0.54	0.818	0.680	0.640

[a] The best classification threshold yielding the highest sum of sensitivity and specificity (investigated from 0.01 to 1.00 with 0.01 intervals).

[b] The performance measures achieved by using the best classification threshold.

Table S6. Predictive performance for the alarm type BEFORE (training day -2 and -3 included) for each of the four data source models (WF: water flow, AF: activation frequency, PTSOLID: pen temperature above the solid floor, PTSLATTED: pen temperature above the slatted floor) and when combined with each other and the model on fixed probabilities (FIXED) through the Bayesian ensemble strategy.

Bayesian ensemble combination	No. of event cases	No. of control cases	AUC	95% CI	Best threshold[a]	Sensitivity[b]	Specificity[b]	Alarm error rate[b]
WF	11	50	0.525	0.334-0.717	0.64	0.455	0.700	0.750
WF + FIXED	11	50	0.627	0.434-0.819	0.45	0.727	0.540	0.742
AF	11	50	0.438	0.255-0.622	0.64	0.364	0.700	0.636
AF + FIXED	11	50	0.513	0.322-0.704	0.65	0.455	0.860	0.583
WF + AF	11	50	0.475	0.287-0.663	0.80	0.273	0.900	0.625
WF + AF + FIXED	11	50	0.584	0.391-0.778	0.72	0.364	0.860	0.636
AF + WF	11	50	0.473	0.285-0.660	0.48	0.727	0.420	0.784
AF + WF + FIXED	11	50	0.549	0.356-0.742	0.38	0.909	0.380	0.756
PTSOLID	12	54	0.581	0.396-0.766	0.68	0.750	0.519	0.743
PTSOLID + FIXED	12	54	0.695	0.517-0.873	0.64	0.750	0.685	0.654
PTSLATTED	12	54	0.444	0.268-0.620	0.83	0.167	0.907	0.714
PTSLATTED + FIXED	12	54	0.574	0.389-0.759	0.43	0.583	0.630	0.741
PTSOLID + PTSLATTED	12	54	0.528	0.344-0.711	0.51	0.750	0.463	0.763
PTSOLID + PTSLATTED + FIXED	12	54	0.611	0.428-0.796	0.46	0.750	0.661	0.700
PTSLATTED + PTSOLID	12	54	0.502	0.320-0.685	0.55	0.750	0.352	0.795
PTSLATTED + PTSOLID + FIXED	12	54	0.603	0.418-0.788	0.67	0.583	0.704	0.696
WF + PTSOLID	11	50	0.570	0.376-0.763	0.81	0.364	0.900	0.556
WF + PTSOLID + FIXED	11	50	0.666	0.476-0.855	0.64	0.636	0.720	0.667
PTSOLID + WF	11	50	0.505	0.315-0.695	0.77	0.455	0.720	0.737
PTSOLID + WF + FIXED	11	50	0.604	0.411-0.797	0.48	0.818	0.440	0.757
WF + AF + PTSOLID + PTSLATTED	11	50	0.468	0.281-0.655	0.92	0.182	0.980	0.333
WF + AF + PTSOLID + PTSLATTED + FIXED	11	50	0.543	0.350-0.735	0.93	0.182	1.000	0.000

[a] The best classification threshold yielding the highest sum of sensitivity and specificity (investigated from 0.01 to 1.00 with 0.01 intervals).

[b] The performance measures achieved by using the best classification threshold.

Table S7. Predictive performance for the alarm type ON (training day -1 included) for each of the four data source models (WF: water flow, AF: activation frequency, PTSOLID: pen temperature above the solid floor, PTSLATTED: pen temperature above the slatted floor) and when combined with each other and the model on fixed probabilities (FIXED) through the Bayesian ensemble strategy.

Bayesian ensemble combination	No. of event cases	No. of control cases	AUC	95% CI	Best threshold[a]	Sensitivity[b]	Specificity[b]	Alarm error rate[b]
WF	12	54	0.500	0.318-0.682	0.27	1.000	0.167	0.789
WF + FIXED	12	54	0.601	0.416-0.786	0.55	0.417	0.852	0.615
AF	12	54	0.510	0.327-0.692	0.85	0.333	0.833	0.692
AF + FIXED	12	54	0.595	0.410-0.780	0.92	0.333	0.944	0.429
WF + AF	12	54	0.529	0.345-0.713	0.44	0.917	0.315	0.771
WF + AF + FIXED	12	54	0.623	0.438-0.807	0.72	0.333	0.556	0.556
AF + WF	12	54	0.441	0.266-0.617	0.94	0.167	0.907	0.714
AF + WF + FIXED	12	54	0.525	0.341-0.708	0.91	0.333	0.889	0.600
PTSOLID	12	54	0.568	0.383-0.753	0.26	0.917	0.264	0.780
PTSOLID + FIXED	12	54	0.652	0.470-0.835	0.18	1.000	0.283	0.760
PTSLATTED	12	53	0.471	0.291-0.650	0.62	1.000	0.208	0.778
PTSLATTED + FIXED	12	53	0.572	0.387-0.758	0.45	1.000	0.208	0.778
PTSOLID + PTSLATTED	12	53	0.594	0.409-0.780	0.63	0.750	0.472	0.757
PTSOLID + PTSLATTED + FIXED	12	53	0.675	0.494-0.856	0.43	1.000	0.340	0.745
PTSLATTED + PTSOLID	12	53	0.525	0.342-0.710	0.58	1.000	0.208	0.778
PTSLATTED + PTSOLID + FIXED	12	53	0.568	0.382-0.753	0.48	1.000	0.226	0.774
WF + PTSOLID	12	53	0.561	0.376-0.746	0.83	0.167	1.000	0.000
WF + PTSOLID + FIXED	12	53	0.607	0.422-0.792	0.68	0.333	0.925	0.500
PTSOLID + WF	12	53	0.513	0.330-0.696	0.47	0.583	0.547	0.774
PTSOLID + WF + FIXED	12	53	0.568	0.383-0.753	0.78	0.250	0.943	0.500
WF + AF + PTSOLID + PTSLATTED	12	53	0.615	0.431-0.800	0.76	0.750	0.509	0.743
WF + AF + PTSOLID + PTSLATTED + FIXED	12	53	0.650	0.467-0.832	0.62	0.833	0.453	0.744

[a] The best classification threshold yielding the highest sum of sensitivity and specificity (investigated from 0.01 to 1.00 with 0.01 intervals).

[b] The performance measures achieved by using the best classification threshold.
